# Influence of Degradation Product Thickness on the Elastic Stiffness of Porous Absorbable Scaffolds Made from an Bioabsorbable Zn–Mg Alloy

**DOI:** 10.3390/ma14206027

**Published:** 2021-10-13

**Authors:** Jannik Bühring, Maximilian Voshage, Johannes Henrich Schleifenbaum, Holger Jahr, Kai-Uwe Schröder

**Affiliations:** 1 Institute of Structural Mechanics and Lightweight Design, RWTH Aachen University, 52062 Aachen, Germany; kai-uwe.schroeder@sla.rwth-aachen.de; 2 Digital Additive Production, RWTH Aachen University, 52074 Aachen, Germany; maximilian.voshage@dap.rwth-aachen.de (M.V.); johannes.henrich.schleifenbaum@dap.rwth-aachen.de (J.H.S.); 3 Institute of Anatomy and Cell Biology, University Hospital, RWTH Aachen University, 52074 Aachen, Germany; hjahr@ukaachen.de

**Keywords:** additive manufacturing, scaffolds, bioabsorbable metals, biodegradation, lattice structures, stiffness properties

## Abstract

For orthopaedic applications, additive manufactured (AM) porous scaffolds made of absorbable metals such as magnesium, zinc or iron are of particular interest. They do not only offer the potential to design and fabricate bio-mimetic or rather bone-equivalent mechanical properties, they also do not need to be removed in further surgery. Located in a physiological environment, scaffolds made of absorbable metals show a decreasing Young’s modulus over time, due to product dissolution. For magnesium-based scaffolds during the first days an increase of the smeared Young’s modulus can be observed, which is mainly attributed to a forming substrate layer of degradation products on the strut surfaces. In this study, the influence of degradation products on the stiffness properties of metallic scaffolds is investigated. For this, analytical calculations and finite-element simulations are performed to study the influence of the substrate layer thickness and Young’s modulus for single struts and for a new scaffold geometry with adapted polar cubic face-centered unit cells with vertical struts (f2cc,z). The finite-element model is further validated by compression tests on AM scaffolds made from Zn1Mg (1 wt% Mg). The results show that even low thicknesses and Young’s moduli of the substrate layer significantly increases the smeared Young’s modulus under axial compression.

## 1. Introduction

The increasingly elderly population and the accompanying rising number of bone fractures have led to a significant rise in physical disabilities. The healing of larger bone defects is still a challenging task in orthopaedics. Using degradable implants eliminates the need for revision surgery, which may be required for some permanent medical devices. Thus, using such implants would not only benefit the patient, but also reduce healthcare costs [1]. Ideally, the implants should present a fully interconnected porous structure and should show equivalent mechanical properties, especially regarding the stiffness [2]. Such a biodegradable bone implant would allow fully natural bone regeneration, while the material gradually disappears in the body through absorption. These requirements can be fulfilled i.e., by additive manufactured (AM) lattice structures. Due to the large number of available materials and design parameters, almost any mechanical and material requirement profile can be set. However, biocompatibility and an interconnected porous structure can be fulfilled by a wide range of materials, reaching equivalent mechanical properties at the same time is still challenging. Biocompatible materials can be found in a wide variety of material classes [3]. One example are polymer-based materials, which offer great advantages in terms of customized biodegradation and design [4]. Further to mention are ceramic materials, which also exhibit the aforementioned biodegradation and offer particularly good healing properties for bone defects [5]. However, for fully load-bearing applications only metals fulfill the needed properties, especially regarding strength and stiffness [6]. The Laser Powder Bed Fusion (LPBF) process enables the individualized production of high-resolution lattice structures with very fine struts (<250 µm) [7,8] at reasonable costs, and is thus ideal for the production of personalized implants [9]. In particular, the use of zinc (Zn), magnesium (Mg), iron (Fe) and their alloys, are increasingly coming into focus for orthopaedic applications [10,11]. Although Fe-based implants would biomechanically, and with respect to their corrosion speed [12,13], gain most from increased porosity [14], their limited cytocompatibility is a concern [15]. Nevertheless, in comparison to pure zinc and magnesium, iron has the highest values regarding yield strength and Young’s modulus ($\sigma_{y,Fe}\approx 200$–352 MPa, $E_{Fe}\approx 188$–215 GPa [16,17,18]; $\sigma_{y,Zn}\approx 12$–32 MPa, $E_{Zn}\approx 43$–150 GPa [12,18,19]; $\sigma_{y,Mg}\approx 51$ MPa, $E_{Mg}\approx 27$–35 GPa [20,21,22]) and offers a large margin for introducing a controlled porosity, which directly influences the strength and stiffness properties of the material. Alloying can further improve the mechanical properties. Adding Zn to Mg-based alloys increases the yield strength and Young’s modulus of the material [3,13,23]. Same goes for Zn alloyed with Mg [3,19,24], whereas adding aluminum to Zn-based alloys leads to a decrease in stiffness and strength [19].

Examples for Mg- and Zn-based studies on porous scaffolds can be found, e.g., in Witte et al. [25], who show the feasibility of producing AM open-porous, biodegradable and biocompatible Mg scaffolds. Li et al. [2] produced AM WE43 (Mg alloy with 4 wt% yttrium and 3 wt% rare earth elements) scaffolds based on diamond unit cells, to show the in vitro biodegradation behavior, mechanical properties and biocompatibility. Furthermore, Kopp et al. [26] showed that the pore size of Mg scaffolds influences the long-term stability, while heat treatment especially effects the degradation and mechanical stability. Cockerill et al. [27] used a casting approach to produce porous structures made of pure Zn and studied the topology, mechanical properties, biodegradation and biocompatibility. Another example is shown by Li et al. [28], who produced scaffolds from Zn with a diamond lattice structure via LPBF and studied the static and dynamic biodegradation behavior.

In a physiological environment biodegradable metals usually show a decreasing Young’s modulus during the degradation process, due to the progressive absorption of the metallic surface, which consequently leads to a reduction of the strut cross section [29,30,31,32]. Since the strut thickness is directly related to the stiffness, the latter will also decrease. Interestingly, during the first days of in vitro corrosion of Mg-based (WE43) scaffolds, an increase of around 40% in the Young’s modulus was recently reported [2]. This increase in stiffness is mainly attributed to the formation of a composite cross section, consisting of the base strut and an adherend layer of degradation products. A brief review of the literature shows [3,10,29,31] that the compound of degradation products, which adheres to the surface of the struts, consists for the most parts of hydroxides, phosphates and carbonates, for which only insufficient mechanical properties can be found. The phosphates and carbonates form a compound of usually unspecified chemical composition that further changes over time. Furthermore, a hydroxide layer is forming on the metallic surface. The basic biochemical processes, responsible for this, can be summarized as followed [29,31,32]:
Anodic reaction$\mathrm{Metal}\to {\mathrm{Metal}}^{n+}+n\left(e^{-}\right)$Cathodic reaction$2H_{2}O+2e^{-}\to 4OH^{-}+H_{2}$$2H_{2}O+O_{2}+4e^{-}\to 4OH^{-}$Product formation${\mathrm{Metal}}^{n+}+n\left(OH^{-}\right)\to \mathrm{Metal}{\left(OH\right)}_{n}$Product dissolution$\mathrm{Metal}{\left(OH\right)}_{n}+2Cl^{-}\to \mathrm{Metal}{\left(Cl\right)}_{2}+2OH^{-}$

**Figure 1:**
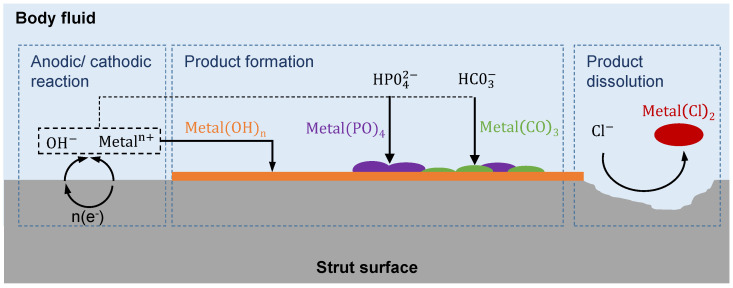
Schematic process sketch of the degradation process of absorbable metals according to Han et al. and Li [29,31].

Figure 1 shows a simplified schematic of the degradation process. The human body fluid releases an anodic reaction, and the free electrons undergo a cathodic reaction under the release of hydrogen and hydroxide ions, which form together with the metal a hydroxide layer on the surfaces of the struts. From equivalent reactions, phosphates and carbonates form on the strut surfaces [29]. These processes are responsible for an increase in stiffness during the early phases of the corrosion process [2]. Later, chloride ions start the dissolution of the biodegradable metal to cause a decrease of the cross-sectional strut diameter of the scaffold.

We now used Zn1Mg (1 wt% Mg) as an example to investigate the influence of degradation products on the elastic stiffness properties of metallic scaffolds using analytical calculations and finite-element (FE) simulations. For this, first, we focused on the direct influence of the forming substrate layer of degradation products on the axial and bending stiffness of single struts. The corroded strut is modeled as a composite beam with a solid Zn1Mg base strut and a thin-walled layer of corrosion products of unspecified chemical composition. Instead of using concretely quantified values for the Young’s modulus for the compound of degradation products, hypothetical multiples of the Zn1Mg Young’s modulus are used. Afterwards, a new scaffold geometry, based on a polar modeling of a f2cc,z unit cell is produced and tested, to validate the FE model. Using the validated model, a FE parametric study is done to investigate the influence of the substrate layer thickness and Young’s modulus of the compound on the smeared Young’s modulus of the scaffold.

## 2. Materials and Methods

### 2.1. Scaffold Manufacturing

The LPBF (Laser Powder Bed Fusion) experiments were performed on an AconityMINI system designed by Aconity3D (Herzogenrath, Germany), which is specifically developed for laboratory use. This system is characterized by an adapted gas flow management to remove the resulting process fume for materials with low melting and evaporating temperature (i.e., zinc: 692 K, 1180 K). These materials tend to produce a large amount of process fume during manufacture. The beam source is a single-mode fiber laser (wavelength of 1064 nm) with up to 400 W of power output. Samples were manufactured on a zinc baseplate using a bidirectional scanning strategy with 90° rotations between consecutive layers. The energy input during exposure was controlled by the selected process parameters (laser power ($P_{L}$), layer thickness ($D_{s}$), scanning speed ($v_{s}$), and hatch distance ($\Delta y_{s}$)). The volume energy density ($E_{V}$) was calculated as followed [33]:

(1) $$E_{V}=\frac{P_{L}}{D_{S}v_{s}\Delta y_{s}}$$

Within the scope of this work, all AM scaffolds were manufactured with a constant layer thickness of 30 µm and $E_{V}$ was set for all scaffolds to 133 J/mm${}^{3}$. The scaffolds were afterwards sandblasted with 2.5 bar, to remove adhering powder particles.

### 2.2. Scaffold Geometry

**Figure 2:**
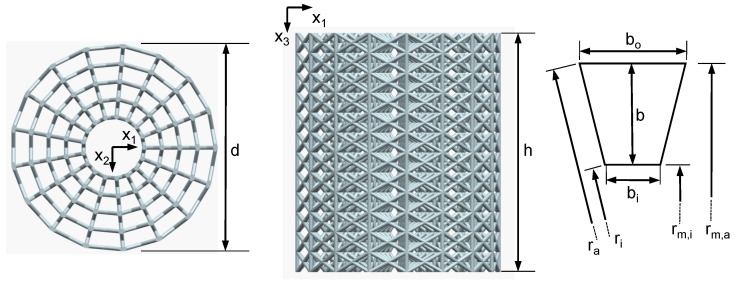
Scaffold geometry used for the study.

Figure 2 shows the scaffold geometry, which was used for the FE study and validation tests. A modified polar f2cc,z unit cell was used. A total number of four cells in radial direction ($n_{1,2}=4$), a total number of 17 cells in circumferential direction (m=17) and a total number of 12 cells in height direction ($n_{3}=12$) was chosen. The scaffold has a total height of h=12 mm and a diameter of d=10 mm. The nominal strut radius is $r_{s}=0.1$ mm. The cell width *b* results from $b=\left(d-2R_{m}\right)/\left(2n_{1,2}\right)=0.9$ mm, where $R_{m}=1.4$ mm is the radius of central cavity, or rather the inner radius of the first cell ring, measured at the cells edges. Since the cells only approximate a circle, the radial position of the midpoint of the cells side faces lies at $r_{m,i}=r_{i}\cos\left(\varphi /2\right)$ for the inner face and $r_{m,a}=r_{a}\cos\left(\varphi /2\right)$ for the outer face, where $r_{i}$ is the inner radius of the cell edges and $r_{a}$ is the outer radius of the cell edges and φ=2π/m is the proportion that a cell has in the total circumference. The strut inclination ω of the circumferential diagonal struts can be calculated for the inner diagonals of each cell ring ($\omega_{i}$) and for the outer diagonals of each cell ring ($\omega_{o}$) as followed:

(2) $$\omega_{i}=\arctan\left(h/b_{i}\right);\omega_{o}=\arctan\left(h/b_{o}\right)$$

**Figure 3:**
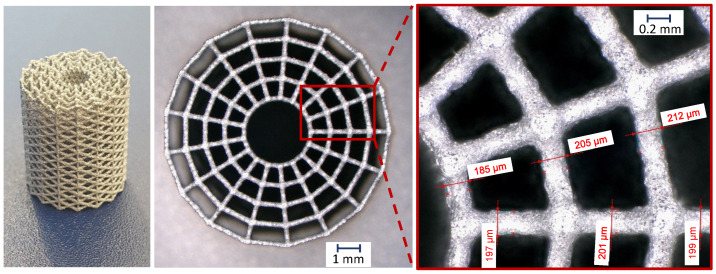
Resulting LPBF produced scaffold used for the physical evaluation.

The radial orientated diagonals strut inclination is equal for all cell rings and results from $\omega_{r}=\tan\left(h/b\right)$. Table 1 sums the resulting geometric parameters of the scaffold. It should be noticed that for the outer rings, the strut inclinations of the diagonals become lower 45°, which usually leads to unfavorable conditions in the AM process. By an optimization of the manufacturing parameters, see Section 2.1 for reference, and the good processability of the material, it was nevertheless possible to produce flat angles, as shown in Figure 3. The strut diameters of the manufactured scaffolds were measured at random positions, resulting in $r_{s}\approx 0.092$–0.106 mm, which lies in an acceptable tolerance range of the nominal strut radius.

### 2.3. Materials and Mechanical Properties

This study focuses a non-commercial Zinc-Magnesium alloy (Zn1Mg—1 wt% Mg), atomized by Nanoval GmbH (Berlin, Germany). The elastic material properties used for the numerical and analytical studies are based on literature data [19,34,35,36]. Validation tests are done on additively manufactured Zn1Mg scaffolds. Furthermore, this study is based on a previous study using Mg-based (WE43) scaffolds [2]. Young’s modulus and yield strength of Zn1Mg were reported by Yang et al. [19]. Young’s modulus of Zn1Mg is documented to be $E_{Zn1Mg}\approx 19$ GPa and yield strength $\sigma_{y,Zn1Mg}\approx 74$ MPa. The mechanical properties for Zn1Mg have been extracted via tensile tests. Both the zinc content as well as the magnesium content will take part in the biochemical reaction process. Material properties for Zn(OH)${}_{2}$, Mg(OH)${}_{2}$, ZnCO${}_{3}$ and MgCO${}_{3}$ from degradation processes are not sufficiently documented in the literature, but can be approximated by extrapolating data i.e., from Ulutan et al. [34], who reported values for the Young’s modulus of Mg(OH)${}_{2}$ of $E_{Mg{\left(OH\right)}_{2}}=64$ GPa, Ulian et al. [35] reporting throughout anisotropic behavior an $E_{Mg{\left(OH\right)}_{2}}\approx 64$–180 GPa, or Yao et al. [36] reporting the Young’s modulus of MgCO${}_{3}$ to be $E_{MgCO_{3}}\approx 150$–260 GPa. For Mg(PO)${}_{4}$ and the degradation products of Zn, insufficient data were found. Due to the poor data concerning material properties and proportions of the composite material, hypothetical Young’s moduli were defined by multiples of the base materials Young’s modulus, which is adequate for the analytical and numerical investigations concerning the general influence.

### 2.4. Analytical Model

**Figure 4:**
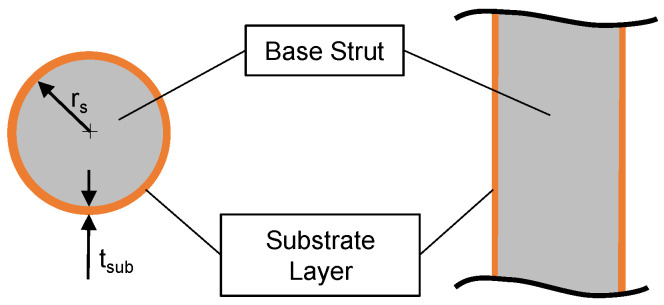
Cross section of the idealized corroded strut; in grey: base strut, in orange: compound of degradation/reaction products.

The metallic strut and the enclosing compound of degradation products can be modeled as a composite beam. Here, the metallic core is surrounded by a thin-walled mineral cross section, which is idealized to be perfectly round in the following, and is demonstrated in Figure 4. Afterwards, the axial and bending stiffness of a composite strut can be calculated by a summation of the individual layer stiffnesses. The resulting equivalent composite axial stiffness $\bar{EA}$ can be calculated as followed:

(3) $$\bar{EA}=\sum E_{j}A_{j}=E_{s}r_{s}^{2}\pi +E_{\mathrm{sub}}\left(2r_{s}t_{\mathrm{sub}}+t_{\mathrm{sub}}^{2}\right)\pi ,$$

where $E_{s}$ is the base materials Young’s modulus, $E_{\mathrm{sub}}$ is the Young’s modulus of the compound of degradation products in the substrate layer, $r_{s}$ is the inner radius of the substrate layer, or rather the base strut radius, and $t_{\mathrm{sub}}$ is the thickness of the substrate layer. For the equivalent composite bending stiffness $\bar{EJ}$ results:

(4) $$\bar{EJ}=\sum E_{j}J_{j}=E_{s}\frac{\pi }{4}r_{s}^{4}+E_{\mathrm{sub}}\frac{\pi }{4}\left({\left(r_{s}+t_{\mathrm{sub}}\right)}^{4}-r_{s}^{4}\right).$$

### 2.5. Finite-Element Model

**Figure 5:**
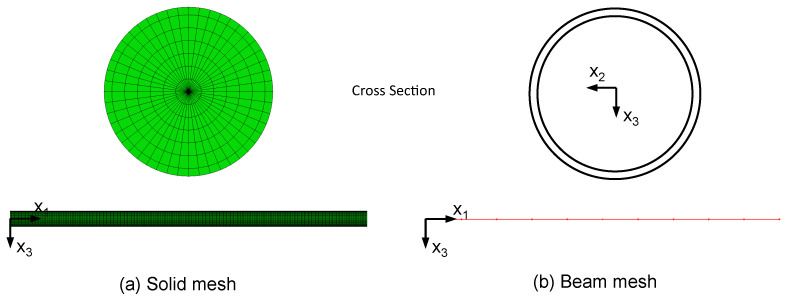
Finite-Element Mesh; (**a**) solid model, (**b**) beam model with schematic cross section.

For the FE calculations Abaqus/Standard with python scripting for model creation was used. The scaffolds were meshed using 3-node quadratic beam elements (B32). A convergence study showed that using five elements per strut gives sufficient results. Linear elastic material behavior and a static, displacement-controlled step was used. A displacement of u=1 mm in axial direction ($x_{3}-$direction) of the scaffold was applied. The summation of the nodal reaction forces in axial direction $F_{3}$ was measured. The resulting stiffness can be calculated from $\left(EA\right)=F_{3}h/u_{3}$. Since for beam elements no composite cross section can be defined in Abaqus/Standard, a generalized beam profile was used. Stiffnesses were defined according to Equations (3) and (4). To validate the beam formulation, a single strut under compression and bending was modeled using (a) a 3D-volume mesh with a hybrid meshing strategy using 10-node quadratic tetrahedron (C3D10) and 20-node quadratic hexagonal (C3D20) elements and (b) the aforementioned beam modeling strategy. For good mesh quality 48 elements in circumferential direction and five elements in radial direction plus one additional element for the substrate layer were used. According to the scaffold mesh, for the single strut beam model a total number of ten 3-node quadratic beam elements (B32) was used. Both models are show in Figure 5. The base strut radius was set to $r_{s}=0.1$ mm and the substrate layer thickness to $t_{sub}=0.01$ mm (see Figure 4 for reference). The strut length is l=5 mm. The beam model cross section was defined with a generalized beam section according to the scaffold model. Struts under both axial compression and bending were examined. For the axial loaded strut, a simply supported beam and for the bending model a cantilever beam model was used.

### 2.6. Compression Testing

**Figure 6:**
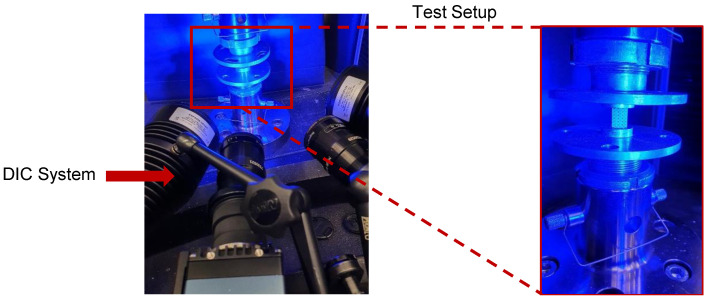
Experimental setup for compression tests on AM Zn1Mg scaffolds.

To validate the FE model, compression tests on equivalent LPBF (Laser Powder Bed Fusion) produced Zn1Mg polar scaffolds were done. A total number of two specimens was tested. The tests were done on an Instron 5567 electric tensile/compression testing machine with 30 kN load cell. The tests were performed displacement controlled with a crosshead speed of $\dot{u}=0.2$ mm/min. The crosshead displacement and load were documented. Since small shifts in the test setup lead to differences between the real and the crosshead displacement, the tests were monitored via DIC-technique (Direct Image Correlation) using an Aramis 4M system by GOM. By this, the real displacement of the specimen can be measured. Figure 6 shows the used setup for the compression tests.

## 3. Results

### 3.1. Analytical Results

**Figure 7:**
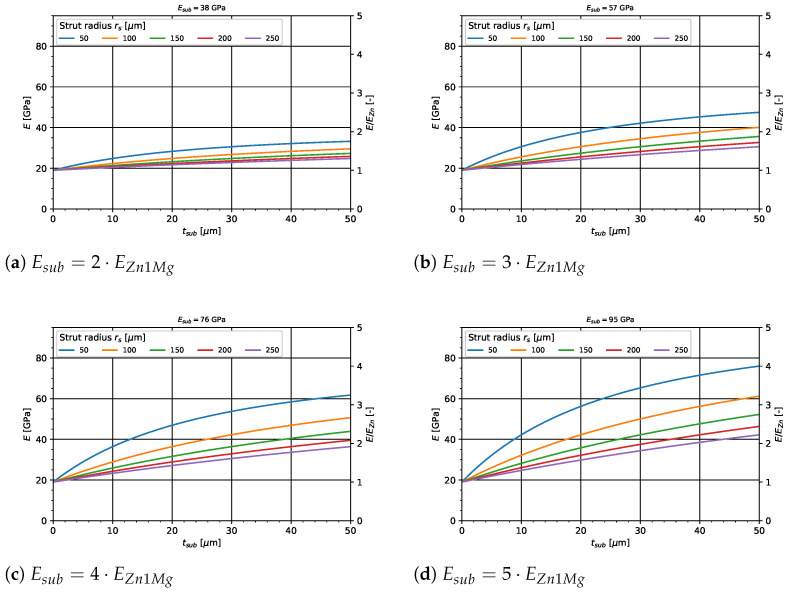
Analytical calculation of the axial stiffness of a single composite strut for varying parameters of the substrate layer.

Figure 7 shows the results of the analytical calculations for the Zn1Mg single struts under axial compression. Shown is resulting composite Young’s modulus *E* as a function of the substrate layer thickness $t_{sub}$ for different strut radii $r_{s}$ (50μm–250μm). Furthermore, Figure 7 (a) shows the resulting absolute composite Young’s modulus (left axis) and relative increase $E/E_{Zn1Mg}$) (right axis) for a Young’s modulus twice as high, (b) three times as high, (c) four times as high and (d) five times as high as the base materials Young’s modulus. It can be noticed that the thinner the struts and the thicker the substrate layer, the higher the resulting composite axial stiffness of the strut. Especially for smaller strut radii, i.e., $r_{s}=50\;\mu$m, as well as for small substrate thicknesses, the effect of an increase in axial stiffness is clearly visible. Same applies for high Young’s moduli of the substrate layer.

**Figure 8:**
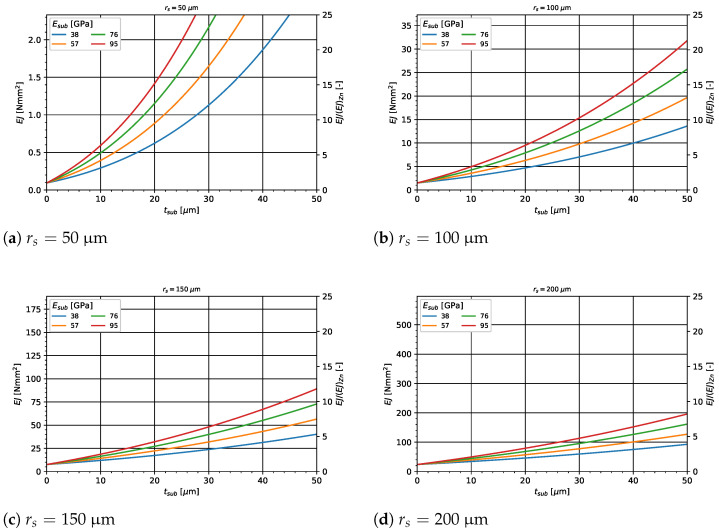
Analytical calculation of the bending stiffness of a single composite strut for varying parameters of the substrate layer.

Figure 8 shows the results for the analytical observations of the Zn1Mg single struts under bending. Shown is the resulting composite bending stiffness EJ as a function of the substrate layer thickness $t_{sub}$ for different substrate Young’s moduli $E_{sub}$, which is set to 2–5 times the base materials Young’s modulus $E_{Zn1Mg}$. Furthermore, Figure 8 shows the resulting absolute composite bending stiffness EJ (left axis) and relative increase $EJ/{\left(EJ\right)}_{Zn1Mg}$) (right axis) for (a) a base strut radius $r_{s}=50\;\mu$m, (b) $r_{s}=100\;\mu$m, (c) $r_{s}=150\;\mu$m and (d) $r_{s}=200\;\mu$m. With increasing substrate layer thickness and higher substrate Young’s modulus, a higher increase in bending stiffness can be observed. Especially for small strut radii, such as $r_{s}=50\;\mu$m, very high increases in bending stiffness can be achieved. This is not only the case for high moduli of the substrate layer, but also in the case when the composite of degradation products has the same Young’s modulus.

### 3.2. Finite-Element Results

#### 3.2.1. Single Strut Simulations

**Figure 9:**
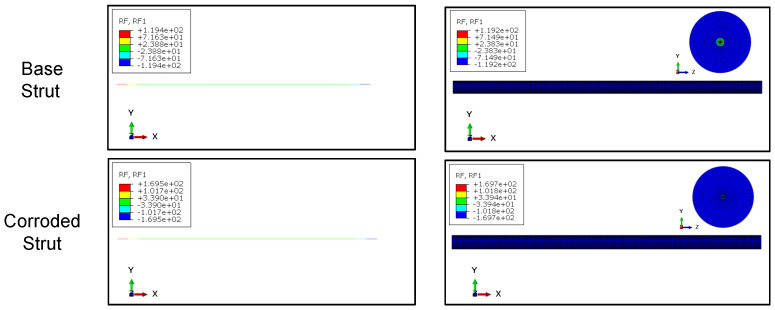
Reaction force (RF) comparison of modeling approaches for base and corroded strut under axial compression; (**left**) beam elements, (**right**) solid elements.

**Figure 10:**
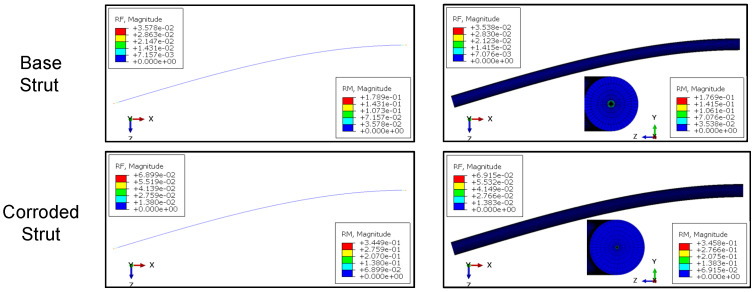
Reaction force (RF) and reaction moment (RM ) comparison of modeling approaches for base and corroded strut under bending; (**left**) beam elements, (**right**) solid elements.

Figure 9 shows the results of the FE simulations of single struts under axial compression. For both the base strut and the corroded strut, the axial reaction force RF1 shows nearly equal values and the difference lies under 0.02%. In Figure 10 the comparison for the bending loaded struts is presented. In both cases the results of the beam model and the solid model are in good agreement. For the base struts, the difference regarding reaction force RF and reaction moment RM lies at around 1%. For the corroded struts, the difference is lower than 0.03%. Furthermore, especially in the case of the corroded strut, the calculation time can be massively decreased using a beam modeling approach.

#### 3.2.2. Whole Scaffold Modeling

**Figure 11:**
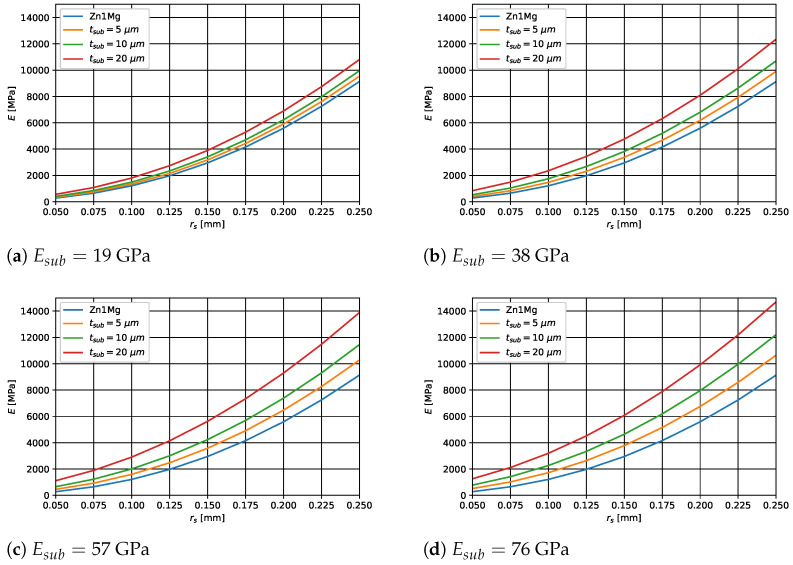
Results of the FE simulations of corroded scaffolds for varying parameters.

Figure 11 shows the results of the FE Scaffold parametric study. Shown is the resulting smeared Young’s modulus *E* of the scaffold, which results from dividing the axial reaction forces by the projected cross section of the whole scaffold *A*, as a function of the base strut radius $r_{s}$ for different thicknesses of the substrate layer $t_{sub}$ and (a) a compound Young’s modulus of the substrate layer of $E_{sub}=19$ GPa (equal to $E_{Zn1Mg}$), (b) $E_{sub}=38$ GPa, (c) $E_{sub}=57$ GPa and (d) $E_{sub}=76$ GPa. The stiffness grows exponentially as a function of the strut diameter and is clearly more pronounced the higher the Young’s modulus of the compound of the substrate. A significant increase in the axial stiffness of the scaffolds can be observed from all hypothetical Young’s moduli of the substrate. Table 2 sums the quantitative results for the respective Young’s moduli. It can be seen that already for a base materials equivalent Young’s modulus of the substrate, small substrate thicknesses of a few microns and small strut radii lead to an increase in stiffness of 22–85%. The effect increases significantly when considering higher layer thicknesses and higher stiffnesses of the substrate layer.

### 3.3. Confirmation by Physical Evaluation

**Figure 12:**
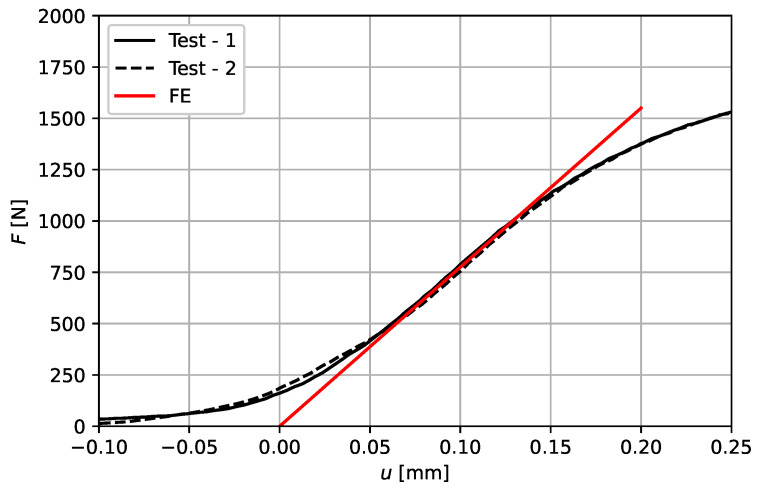
Validation of FE model: Resulting load-displacement curve of two tested LPBF produced scaffolds and equivalent FE model.

Figure 12 shows the results of the two tested scaffolds under axial compression in comparison to the FE result. The tests show reproducible behavior regarding the stiffness. The smeared Young’s modulus of the scaffolds can be calculated in the linear region of the load-displacement curves by E=Fh/(Au), where *F* is the measured force in the machines load cell, *h* is the total height of the scaffold, *A* is the projected smeared cross section of the scaffold and *u* is the displacement associated with the measured force. From the tests, a Youngs’s modulus of approximately $E_{test}\approx 1125$ MPa can be determined, measured in the area between 600–800 N. From the FE model a Young’s modulus of $E_{FE}=1258$ MPa can be extracted. Furthermore, the FE model shows that for loads smaller 800 N, nowhere the strut-stresses have exceeded the yield point. The slight differences could be attributed to local deviations in the strut diameter of the AM scaffolds, as shown in Section 2.2 respectively Figure 3. Furthermore, the modeling using beam elements neglects the accumulation of material in the nodes of the real scaffold. Furthermore, the used Young’s modulus is based on literature data and it is well known that Young’s moduli of AM materials tend to show slight differences (see also Section 1). Nevertheless, the tests show that the FE model based on beam elements provides sufficiently accurate results in terms of the resulting smeared axial stiffness and can be used for the parametric study.

## 4. Discussion

We investigated the influence of degradation products on the elastic stiffness properties of biodegradable metallic scaffolds. For this, a hypothetical compound of degradation products was modeled as a thin-walled layer with a homogeneous cross section. The compound of degradation products consists for the most parts of hydroxides, phosphates and carbonates [29,30,31,32]. Since there is no sufficient database, yet, for the mechanical properties of the degradation products, hypothetical Young’s moduli were defined using multiples of the Young’s modulus of the base material, which was obtained from literature data [3,12,13,16,17,18,19,20,21,22,23,24]. By this, the influence of the degradation products on the elastic stiffness properties as a function of the layer thickness and Young’s modulus could be investigated. This was done using analytical models and finite-element simulations for single struts, to show the direct influence of the layer of degradation products on the axial and bending stiffness, as well as for whole scaffold geometries, to show the superposed influence on the axial smeared Young’s modulus of a specific scaffold geometry. Two modeling approaches were contrasted for the FE simulations, first a meshing strategy using a 3D volume mesh and second using beam elements. Both approaches show concurring results. For this reason, the beam model was used for a parametric study on whole lattice scaffold geometries, due to the enormous difference regarding the calculation time. To validate the FE model, scaffolds were produced via LPBF and compression tests on two scaffolds were done.

From the single strut investigations can be concluded that depending on the substrates Young’s modulus and the ratio of strut radius to thickness of the substrate layer, significant increases of the composite axial and bending stiffness is expected. The effect intensifies, the smaller the base strut radius in the initial state is. This applies as well as for relatively low Young’s moduli of the substrate layer as for very high Young’s moduli. In comparable studies [2,14,15,25,26], mentioned in the introduction part, strut diameters of 300–400 µm were used for orthopaedic scaffolds. Even for low layer thicknesses (i.e., 10 µm) and low Young’s moduli, for single struts with diameters in this range, depending on the thickness of the substrate layer and the composite module, an increase of more than 10% for the Young’s modulus under axial compression and more than 40% in bending stiffness can be expected, which is not to be confused with the Young’s modulus in the bending load case. To validate the base FE model, physical test results were compared to an equivalent FE simulation, using beam elements for meshing. As presented in the results section, the beam modeling shows similar results, compared to a much more numerically expensive meshing strategy with solid elements. The compression tests on LPBF produced scaffolds show reproducible results and furthermore equivalent smeared Young’s moduli in the FE model and physical tests. For this reason, a FE parametric study on the tested geometry was done by varying the substrate layer thickness and the Young’s moduli of the compound of the degradation products in the substrate layer, to study the influence of the substrate layer on the smeared Young’s modulus of complex scaffold geometries. Our results show that an enormous increase in stiffness can be expected even for complex geometries, which was also observed by Li et al. [2] for diamond lattice structures made from WE43. For the previously mentioned example of strut diameters of 300–400 µm, the investigations on the scaffolds show that a much stronger effect can be observed due to the superposition of the axial and bending stiffness increase. As presented in the results section, the increase of the smeared axial Young’s modulus under compression can be quantified to approximately 10–40% for a layer thickness of 10 µm and varying Young’s moduli. The effect intensifies to values of approximately 20–80%, if i.e., a layer thickness of 20 µm is assumed. From this can be followed that compared to the separated reflection of the influence of the substrate layer on axial and bending stiffness of single struts, the effect of a stiffness increase is clearly more pronounced in the case of scaffold geometries. This is mainly attributable to the combined loading in compression and bending of the struts, which both ultimately have a direct effect on the smeared Young’s modulus of the scaffold. Nevertheless, the investigations on single struts give clear indications about the formation of the effect. Furthermore, the analytical expressions show the direct influence of the thickness and Young’s modulus of the degradation products.

## 5. Conclusions

In conclusion, our analytical and numerical modeling approach basically confirmed earlier assumptions by Li et al. [2] that the increase in stiffness of corrosion product layer-coated AM WE43 is indeed due to formation of a composite beam of base strut and substrate layer. As shown in this discussion, even for low thicknesses and Young’s moduli of the degradation product layer, axial stiffness increases of more than 40% can be achieved. Even though the geometry of the scaffold is different at the investigations of Li et al., this study clearly shows the influence on the stiffness. Nevertheless, our results must be validated by further investigations on corroded single struts or equal, to validate the formation of an almost homogeneous layer of degradation products and to obtain more knowledge about the real composite Young’s modulus, or rather the Young’s modulus of the compound of degradation products.

## Figures and Tables

**Table 1:** Resulting geometric parameters of the scaffold used for this study ($R_{m}=1.4$ mm); *i* defines the actual ring, starting from the middle with i=1 according to Figure 2.

*i*	$r_{i}$ [mm]	$r_{m,i}$ [mm]	$b_{i}$ [mm]	$\omega_{i}$ [${}^{\circ }$]	$r_{o}$ [mm]	$r_{m,o}$ [mm]	$b_{o}$ [mm]	$\omega_{o}$ [${}^{\circ }$]
1	1.4	1.376	0.515	62.8	2.3	2.261	0.845	49.8
2	2.3	2.261	0.845	49.8	3.2	3.146	1.176	40.4
3	3.2	3.146	1.176	40.4	4.1	4.030	1.507	33.6
4	4.1	4.030	1.507	33.6	5.0	4.917	1.838	28.6

**Table 2:** Percentage increase of the smeared Young’s modulus *E* for varying substrate Young’s moduli $E_{sub}$ and layer thicknesses $t_{sub}$.

$E_{\mathrm{sub}}$ [GPa]	$t_{\mathrm{sub}}$ [µm]	$r_{s}$ [mm]				
		0.05	0.1	0.15	0.2	0.25
19	5	22%	11%	8%	6%	4%
	10	46%	23%	15%	11%	9%
	20	102%	49%	35%	23%	18%
38	5	43%	22%	14%	11%	8%
	10	91%	44%	29%	22%	17%
	20	201%	95%	61%	45%	35%
57	5	64%	32%	21%	16%	13%
	10	136%	66%	43%	32%	25%
	20	300%	140%	90%	66%	52%
76	5	85%	42%	28%	21%	17%
	10	180%	87%	57%	42%	34%
	20	353%	164%	106%	78%	61%

## Data Availability

The data presented in this study are available on request from the corresponding author.
